# Diversifizierung der Rauchentwöhnungsprogramme – die Rolle der E-Zigarette

**DOI:** 10.1007/s00103-021-03435-5

**Published:** 2021-10-12

**Authors:** Heino Stöver

**Affiliations:** Institut für Suchtforschung Frankfurt (ISFF), Frankfurt University of Applied Sciences, Nibelungenplatz 1, 60318 Frankfurt am Main, Deutschland

**Keywords:** Sucht, Tabakkontrolle, Harm Reduction, Nikotinersatz, Addiction, Tobacco control, Harm reduction, Nicotine replacement

## Abstract

Ob E‑Zigaretten ein nützliches Werkzeug zur Rauchentwöhnung sind, ist in der Wissenschaft bis heute hoch umstritten. In der Realität steigen aber kontinuierlich die Zahlen derer, die mithilfe dieses Produktes versuchen, sich den Tabakkonsum abzugewöhnen. Dieser Diskussionsbeitrag gibt einen Überblick über die aktuelle Forschung zur Frage, ob und inwiefern E‑Zigaretten tatsächlich beim Rauchausstieg helfen können.

Zwar besitzt die E‑Zigarette auch ein gewisses Schadenspotenzial, insbesondere dann, wenn der Nikotinkonsum unnötig verlängert wird. Dennoch kann sie einen wichtigen Beitrag zur Schadensminimierung bei einer Nikotinabhängigkeit leisten. Daher sollte das Produkt auch in der wissenschaftlichen Diskussion als eine ernsthafte Alternative zu Nikotinersatztherapien (NET) behandelt werden. Denn die E‑Zigarette bietet eine weniger schädliche Aufnahmeform für Nikotin an, die viele gesundheitliche Risiken des Tabakkonsums wie die Aufnahme karzinogener Stoffe stark reduziert. Aktuelle Studien und Übersichtsarbeiten deuten darauf hin, dass das Produkt mindestens genauso effektiv helfen kann wie NET. Einzelne Studien weisen sogar eine erhöhte Effektivität der E‑Zigarette bei der Unterstützung eines Rauchausstiegs nach.

Nichtsdestotrotz zeigt die Beschäftigung mit der aktuellen Literatur, dass weitere hochwertige Forschung notwendig ist, um das Produkt und seine Eigenschaften noch besser zu verstehen. Im Sinne des Ansatzes der Harm Reduction (Schadensminderung) wäre es allerdings schon heute vernünftiger, auch auf die E‑Zigarette zu setzen, anstatt die sofortige und vollständige Nikotinabstinenz erzwingen zu wollen. Denn beim Prozess des Rauchausstiegs ist eine schnellstmögliche Beendigung des stärker gesundheitsschädlichen Konsums von Tabak zu priorisieren, um unnötige gesundheitliche Risiken umgehend zu minimieren.

## Abstract (en)

The question of whether e‑cigarettes are a useful tool for smoking cessation is part of an ongoing and highly controversial scientific debate. In practice, however, the number of people trying to quit tobacco consumption by using these products is steadily increasing. This discussion article provides an outline of the current state of research on the issue of whether and to what extent e‑cigarettes can help people quit smoking.

Of course, e‑cigarettes also have a certain potential for harm, especially if nicotine dependence is unnecessarily prolonged. Nevertheless, it can make an important contribution to harm reduction in nicotine consumption. Hence, the product should get treated as a serious alternative to nicotine replacement therapies (NRTs) in the scientific discussion. Like NRTs, e‑cigarettes offer a less harmful way of consuming nicotine that greatly reduces many of the health risks associated with tobacco consumption, such as the absorption of carcinogenic substances. Recent studies and reviews suggest that the product can be at least as effective as nicotine replacement therapies in helping people quit smoking. Individual studies even demonstrate the increased effectiveness of e‑cigarettes in supporting smoking cessation.

Nevertheless, the review of the current literature shows that more high-quality research is needed to further understand the product and its properties. In terms of the harm reduction approach, however, it would already be more appropriate to also rely on e‑cigarettes instead of trying to force immediate and complete nicotine abstinence. After all, in the process of quitting smoking, the fastest possible cessation of the more unhealthy consumption of tobacco should be prioritized in order to immediately minimize unnecessary health risks.

## Einleitung

Das Rauchen von Tabak ist die Ursache einer Vielzahl von Krankheiten, wie Lungenkrebs, COPD (Chronic Obstructive Pulmonary Disease; Sammelbegriff für eine chronisch obstruktive Bronchitis und Emphysem)^1^ oder Schlaganfälle. Damit ist Rauchen das bedeutendste einzelne und vermeidbare Gesundheitsrisiko sowie die führende Ursache frühzeitiger Sterblichkeit [1]. Aufgrund der Vielzahl der negativen gesundheitlichen Auswirkungen des Rauchens gibt es seit Jahrzehnten unter Medizinern, Therapeuten und in der (Gesundheits‑)Politik Bestrebungen, diese Art des Nikotinkonsums durch weniger schädliche Formen der Nikotinaufnahme zu ersetzen [2].

In diesem Zusammenhang hat es auch die E‑Zigarette als verhältnismäßig neues Produkt in den öffentlichen und wissenschaftlichen Diskurs geschafft. Eine der am häufigsten erörterten Fragen hierbei ist, inwiefern sie zur Rauchentwöhnung geeignet ist und wie sie die Erreichung von Public-Health-Zielen, wie unter anderem die Senkung der Raucherrate und die Vermeidung von tabakassoziierten Krankheiten, unterstützen kann [3]. Diese Diskussion wird nicht nur in der wissenschaftlichen Fachwelt geführt. Der intensive Diskurs über das Produkt und seinen möglichen Nutzen hat schon seit einiger Zeit auch Eingang in die Medienwelt gefunden.^2^^,^^3^^,^^4^^,^^5^

Dieser Artikel will einen Überblick über die Chancen und Risiken der E‑Zigarette geben. Nach einer Schilderung des aktuellen Stands des Themas Rauchausstieg in Deutschland wird die Eignung der E‑Zigarette als Ausstiegsprodukt zur Schadensminderung (Harm Reduction) als eine der beiden zentralen Dimensionen der aktuellen Debatte diskutiert. Hierzu werden die aktuellen wissenschaftlichen Erkenntnisse hinsichtlich des Harm-Reduction-Potenzials erörtert und kritisch eingeordnet. Danach widmet sich der Beitrag der Eignung der E‑Zigarette zum Rauchstopp und damit der zweiten wichtigen Dimension. Hier wird vor allem die Vergleichbarkeit von E‑Zigaretten mit anderen nikotinhaltigen Ausstiegsprodukten wie Nikotinersatztherapien (NET) diskutiert werden.

## Aktueller Stand des Themas Rauchausstieg in Deutschland

Deutschland bleibt im Hinblick auf den Tabakkonsum weiterhin ein Hochkonsumland. Jüngste Zahlen zeigen, dass zwar in den vergangenen Jahren ein Präventionserfolg erzielt werden konnte und die Konsumentenzahlen kontinuierlich sinken [4], doch im Vergleich zu anderen europäischen Staaten befinden sich die Zahlen weiterhin mindestens auf einem mittleren Niveau [5].

Aktuelle Schätzungen beziffern den volkswirtschaftlichen Schaden, der durch Tabakkonsum in Deutschland entsteht, auf etwa 97 Mrd. € pro Jahr. Das überwiegt bei Weitem die steuerlichen Einnahmen, die durch die Besteuerung von Tabakprodukten generiert werden können [6]. Gleichzeitig steigt aber auch in der Bevölkerung der Bedarf an Rauchausstiegsprogrammen. So haben laut aktuellen Erhebungen 53 % der aktuellen Raucher in der Europäischen Union (EU) jemals einen Rauchausstieg unternommen [5].

Das Bewusstsein für die Notwendigkeit von evidenzbasierten Rauchausstiegsprogrammen ist dabei bei Medizinern wie auch Therapeuten schon seit Jahren vorhanden. Neben Maßnahmen wie Kurzberatung, Verhaltenstherapie, verschreibungspflichtigen Medikamenten oder Telefonberatung ist auch eine Vielzahl von verschiedenen Nikotinersatzpräparaten, sogenannte Nikotinersatztherapien (NET), länger auf dem deutschen Markt erhältlich. In der wissenschaftlichen Praxis wird akzeptiert, dass die Nikotinquelle Tabakzigarette zeitlich befristet durch eine weniger schädliche Nikotinquelle wie Pflaster oder Kaugummis ersetzt wird [7]. Der zentrale Fokus dieser in der S3-Leitlinie zu Rauchen und Tabakabhängigkeit empfohlenen Therapieform liegt dabei auf der Unterstützung des Rauchausstiegs durch die Linderung von Entzugssymptomen. Beim Ziel der Harm Reduction bei nicht ausstiegswilligen Rauchern werden auch Nikotinersatzprodukte empfohlen, zu denen geringere Evidenz vorliegt [8]. Verschiedene Studien stützen dieses Verfahren. So zeigen Untersuchungen mit unterschiedlichsten Forschungsdesigns und Versuchsgruppen, dass NET durchaus effektiv sind und den Patienten beim Rauchstopp helfen [7]. Trotz der auf wissenschaftlicher Evidenz basierenden Empfehlung zur Nutzung dieser Behandlungsmethode scheint sie bei Rauchern, zumindest in Deutschland, bisher nicht besonders beliebt zu sein [4].

Im Gegensatz dazu haben die Konsumentenzahlen bei der E‑Zigarette in den vergangenen Jahren kontinuierlich zugenommen – insbesondere im Kontext eines Rauchausstiegsversuchs [9]. In der Veröffentlichung der ersten Ergebnisse der Deutschen Befragung zum Rauchverhalten (DEBRA-Studie) gab knapp jeder zehnte Befragte an, mit der E‑Zigarette (nikotinfrei sowie nikotinhaltig) einen Versuch des Rauchausstiegs unternommen zu haben. Andere Hilfsmittel zur Entwöhnung werden deutlich seltener in Anspruch genommen [4]. Auch europaweit geht der Trend bei Konsumenten dahin, mit der E‑Zigarette die Entwöhnung vom Tabakkonsum zu versuchen [10]. Gleichzeitig zeigt sich sowohl auf internationaler als auch auf deutscher Ebene, dass NET weniger stark von ausstiegswilligen Konsumenten nachgefragt werden [4, 11] und die Mehrheit aller Rauchausstiegsversuche generell ohne unterstützende Programme oder Produkte durchgeführt wird [12]. Das ist dahin gehend dramatisch, als dass nahezu 95 % dieser nicht assistierten Versuche innerhalb eines Jahres erfolglos bleiben [4].

## Die E-Zigarette als Nikotinersatzprodukt zur Schadensminderung

In der wissenschaftlichen Diskussion hat sich anhand verschiedener Punkte ein Dissens entwickelt, ob E‑Zigaretten nun für einen Rauchausstieg geeignet sind oder nicht. Trotz ihrer zunehmenden Anwendung bei Rauchstoppversuchen [4] gibt es aktuell noch keinen wissenschaftlichen Beleg, dass sie tatsächlich vermehrt zu einer Tabakabstinenz führen [13]. Um einen langfristig erfolgreichen Rauchstopp zu erreichen, erscheint es jedoch sinnvoll, in einem ersten Schritt die bisherige Nikotinquelle (in den meisten Fällen die Tabakzigarette) durch eine weniger schädliche Quelle (z. B. E‑Zigarette) zu ersetzen, anstatt eine unmittelbare Tabak- und Nikotinabstinenz anzustreben. Durch diese Vorgehensweise soll die gesundheitliche Belastung durch die Verbrennungsstoffe der Tabakzigarette umgehend reduziert werden [14]. Nach dieser Handlungslogik werden auch Nikotinpflaster oder Nikotinkaugummis zeitlich begrenzt verschrieben [7]: Nicht der Rauchstopp, sondern die Schadensminderung ist dabei das erste Behandlungsziel.

Grundsätzlich kann auch die E‑Zigarette auf diese Art und Weise funktionieren: Sie ersetzt die schädliche Nikotinaufnahme in Form der Tabakzigarette durch eine weniger schädliche Nikotinquelle [15]. Auch sie könnte in einem klar definierten Zeitraum und innerhalb eines fest definierten Verfahrens eingesetzt werden. Bisher wird eine Therapie mit der E‑Zigarette aber noch nicht empfohlen [8].

Die Befürworter der E‑Zigarette argumentieren damit, dass das Produkt eine hohe Ähnlichkeit in der Anwendung und bei der Handhabung zu einer herkömmlichen Zigarette hat und es dabei gleichzeitig schaffe, deutlich weniger Schadstoffe auszustoßen [16]. Auch die Rituale des Rauchens können bei weniger Schadstoffaufnahme in Teilen übernommen werden. [17]. Dies wird allerdings auch teilweise kritisch gesehen, da durch die ähnliche Haptik eine vollständige Nikotinentwöhnung schwieriger werden könnte [17]. Es besteht die Befürchtung, dass der Nikotinkonsum nur reduziert, aber nicht komplett aufgegeben wird [18].

Aktuell ist es jedoch noch unklar, welche gesundheitlichen Langzeitfolgen aus dem dauerhaften Konsum von E‑Zigaretten in Hinblick auf ihren Nikotingehalt resultieren [7]. Zwei Metastudien zeigen auf, dass der langfristige Nikotinkonsum mit diversen negativen gesundheitlichen Effekten assoziiert ist [19, 20].

Neben dem weiteren Konsum von Nikotin wird auch die fortgesetzte Schadstoffexposition in Form von Aerosolen kritisch gesehen. Es herrscht zwar wissenschaftlicher Konsens, dass die Nutzer von E‑Zigaretten sich weniger Schadstoffen aussetzen als Tabakraucher [16]. Aber auch hier sind die Langzeiteffekte noch nicht ausreichend untersucht und verstanden worden [16]. Die bisherigen Erkenntnisse am Menschen und am Tier deuten zumindest darauf hin, dass auch durch die Aerosole weiterhin eine gewisse Gesundheitsgefahr ausgehen kann [21].

Als weiteren Aspekt führen die Kritiker von E‑Zigaretten den sogenannten Dual-use an. Beim Dual-use werden von den Nutzern zwar E‑Zigaretten konsumiert, aber parallel weiterhin Tabakzigaretten geraucht [22]. Im Jahr 2018 praktizierten 74,5 % der E‑Zigarettennutzer diese Form des Doppelkonsums [23]. Shahab et al. [18] stellen fest, dass nur der konsequente Umstieg auf E‑Zigaretten oder Nikotinersatzprodukte langfristig mit einer Senkung von krebserregenden oder anderen giftigen Substanzen im Organismus einhergeht. Gleichzeitig blieb die Nikotinaufnahme bei Langzeitnutzern von E‑Zigaretten oder Nikotinersatzprodukten bei allen Gruppen auf einem vergleichbaren Niveau [18]. Auch deuten die Ergebnisse von 2 Kohortenstudien darauf hin, dass ein kompletter Rauchstopp bei Dual-Usern weniger wahrscheinlich ist als bei Personen, die ausschließlich die E‑Zigarette benutzen [13]. Sollte sich dies bestätigen, würde dies ebenfalls gegen die Hypothese des langfristigen dualen Konsums sprechen. Aktuelle Studien zeigen jedoch auch, dass sich der Konsum von E‑Zigaretten reduzierend auf die Anzahl der konsumierten Tabakzigaretten auswirkt [24]: Dual-User konsumieren also weniger Tabakzigaretten als vorher.

Dennoch sollte weiterhin die Prämisse gelten, dass die vollständige Abstinenz des Tabakkonsums schnellstmöglich erreicht werden sollte. Denn insbesondere bei den gesundheitlichen Auswirkungen des Dual-use ist die Forschungslage aktuell noch sehr uneindeutig. So gibt es Studien, die darauf hindeuten, dass Dual-User weniger toxische Substanzen aufnehmen als ausschließlich Tabak konsumierende Probanden [7]. Andere Studien deuten allerdings in die genau entgegengesetzte Richtung [25]. Dementsprechend muss ein vollständiger Umstieg schnellstmöglich erreicht werden, da bisher keine definitiven Aussagen über die gesundheitlichen Effekte dieser Konsumform getroffen werden können [7].

Bei der Diskussion um diesen Aspekt wird allerdings häufig vernachlässigt, dass es auch einen (zeitlich begrenzten) Dual-use bei NET gibt. Interessanterweise ist dieser langfristig mit einem erhöhten Rauchausstieg verbunden [26]. Somit lässt sich festhalten, dass Dual-use ein weitverbreitetes Phänomen beim Rauchausstieg ist und auch bei NET vorkommt. Deswegen kann dieser Aspekt nicht per se gegen die E‑Zigarette verwendet werden – insbesondere dann nicht, wenn das langfristig gewünschte Ziel der vollständigen Tabakabstinenz nichtsdestotrotz erreicht wird.

Es lässt sich festhalten, dass die E‑Zigarette durchaus das Potenzial besitzt, als Instrument zur Schadensminderung eingesetzt zu werden. Ähnlich wie bei etablierten NET (z. B. dem Nikotinpflaster) wird dabei die schnellstmögliche Tabakabstinenz gegenüber der Nikotinabstinenz in der Behandlung priorisiert. Das Ziel ist es, die Schadstoffbelastung im Körper zu verringern. Es muss jedoch beachtet werden, dass die E‑Zigarette nicht schadstofflos ist und auch hier negative gesundheitliche Effekte möglich sind. Zudem muss der aktuell noch zu oft praktizierte Dual-use schnellstmöglich beendet werden, um die bestmögliche Schadensminimierung zu erreichen.

Die Frage, ob das vorhandene Potenzial der E‑Zigarette als Rauchentwöhnungsmittel auch tatsächlich von Nutzern abgerufen werden kann, wird Gegenstand des nächsten Abschnitts sein. Auch der notwendige Vergleich zur Effektivität von etablierten NET soll in diesem Zusammenhang diskutiert werden.

## Effektivität der E-Zigarette bei der Tabakentwöhnung

Schon 2013 wurde die Effektivität der E‑Zigarette bei der Rauchentwöhnung untersucht [27]. Diese Untersuchung zeigte auf, dass E‑Zigaretten sowohl kurzfristig als auch langfristig den Rauchstopp unterstützten und gleichzeitig keine schwerwiegenden Nebenwirkungen bei den Konsumenten auftraten. Zwar konnte diese Studie aufgrund ihres Designs nicht abschließend untersuchen, wie sich die Zahlen relativ zu anderen NET verhalten, doch die Ergebnisse deuten darauf hin, dass die E‑Zigarette ähnlich effektiv wie andere Rauchausstiegsprodukte ist. Ähnliche Studienergebnisse konnten Bullen et al. [26] für ihre Untersuchung in Neuseeland berichten. Ebenfalls deuten die Befunde des Cochrane-Reviews [7] darauf hin, dass nikotinhaltige E‑Zigaretten hinsichtlich einer Unterstützung des Rauchausstiegs vermutlich so effektiv sind wie NET. Aber nicht nur die Möglichkeit eines erfolgreichen Rauchausstiegs steigt mit der Nutzung der E‑Zigarette, sondern auch die Wahrscheinlichkeit, überhaupt einen Rauchstoppversuch zu unternehmen [28]. Letztendlich führt der Gebrauch einer E‑Zigarette dazu, dass mehr Raucher einen Rauchausstieg versuchen und dieser auch in einer mindestens dreimonatigen Tabakabstinenz mündet [28].

Eine Analyse der Daten des National Health Interview Survey (NHIS; [29]) für die USA bestätigt den vorher beschriebenen Trend, wonach die E‑Zigarette immer häufiger für den Rauchausstieg genutzt wird. Die Studienergebnisse zeigen, dass der Konsum der E‑Zigarette positiv mit dem Versuch korreliert, einen Rauchstopp in den vergangenen 12 Monaten erreicht zu haben. Auch eine höhere Tabakabstinenz ist positiv mit der Nutzung des Produktes verbunden [29]. Zudem zeigt sich, dass Nutzer, die sowohl den Tabakkonsum als auch den Konsum der E‑Zigarette überwunden haben, ein sehr geringes Interesse haben, wieder mit der E‑Zigarette anzufangen. Ferner plant die Mehrheit der aktuellen Nutzer einen zeitnahen Ausstieg aus dem E‑Zigarettenkonsum [30].

Doch nicht alle Raucher sind von der E‑Zigarette als Möglichkeit zum Rauchausstieg überzeugt. So konnte eine Studie am Beispiel Nordenglands aufzeigen [31], dass viele Raucher auf den Umstieg zur E‑Zigarette verzichteten. Ihr Motiv dafür war, dass sie befürchteten, so die eine Sucht (Tabakzigaretten) durch eine andere Sucht (E-Zigaretten) zu ersetzen. Es zeigt sich, dass E‑Zigaretten von der Zielgruppe in Teilen selbst skeptisch gesehen werden.

Eisenberg et al. [32] untersuchen ebenfalls, inwiefern die E‑Zigarette beim Rauchausstieg hilft. Trotz der methodischen Defizite im Design (keine ausreichende Bereitstellung von E‑Zigaretten durch den Hersteller in der zweiten Erhebungsphase, damit verbunden Rekrutierungsstopp und Laufzeitverkürzung der Studie) gibt diese Untersuchung interessante Hinweise auf die Effektivität der E‑Zigarette als Rauchausstiegsprodukt. So deuten die Ergebnisse darauf hin, dass die Verhaltenstherapie in Kombination mit der E‑Zigarette innerhalb der ersten 12 Wochen deutlich effektiver darin ist, den Rauchstopp zu fördern, als eine therapeutische Unterstützung allein. Bei längerer Anwendung nivelliere sich aber dieser Effekt und die E‑Zigarette machte keinen signifikanten Unterschied mehr aus. Dieses Ergebnis legt nahe, dass die E‑Zigarette zumindest zu Beginn eines Rauchausstiegs angewendet werden sollte. Allerdings muss diese Erkenntnis dahin gehend eingeschränkt werden, dass in den zweiten 12 Wochen des Studienverlaufs ein Teil der Probanden aufgrund der oben beschriebenen Herstellerprobleme eigene E‑Zigaretten verwendete, was eventuell mit der geringen Wirkung in der zweiten Erhebungsphase in Zusammenhang steht.

Die Ergebnisse von Eisenberg et al. [32] werden durch Levy et al. [33] mithilfe einer Modellierung auf Basis von US-Gesundheitsdaten unterstützt, auch wenn die Grundannahmen der Modellierung durchaus optimistisch gewählt sind. Den Ergebnissen dieser Studie zufolge zeigt sich, dass der verstärkte Einsatz von E‑Zigaretten und anderen neuartigen Nikotinprodukten im Public-Health-Kontext einen signifikanten positiven Effekt auf die Raucherzahlen und die gesundheitlichen Folgen, die mit dem Rauchen assoziiert sind, haben kann. Die verschiedenen durchgeführten Rechnungen, mit jeweils leicht abgeänderten Modellen, weisen für die unterschiedlichen Kohorten in allen Fällen eine deutliche Verringerung der vorzeitigen Todesfälle aufgrund des signifikanten Rückgangs des Tabakkonsums auf. Auf Basis dieser Modellrechnungen lässt sich zudem festhalten, dass auch andere Nikotinquellen wie E‑Zigaretten oder Tabakerhitzer einen starken positiven Effekt bei der Rauchentwöhnung haben können. Es wäre daher klug, diese vermehrt einzusetzen. Denn so werden nicht nur langfristig Kosten im Gesundheitssystem gesenkt, sondern auch tatsächlich Menschenleben geschützt. Die aktuelle Literatur deutet zudem darauf hin, dass E‑Zigarettennutzer sukzessive den Nikotingehalt ihres E‑Zigarettenliquids senken [31, 34, 35] und somit langfristig ihre Nikotinexposition verringern.

Allerdings weisen einzelne Studien auch darauf hin, dass der reduzierte Nikotingehalt durch erhöhtes Dampfen ausgeglichen wird [35], wodurch keine lineare Senkung der Nikotinaufnahme erreicht wird. Gleichzeitig verursacht dies eine erhöhte Exposition gegenüber im Dampf enthaltenen Schadstoffen. Das sind keine idealtypischen Entwicklungen, doch merken auch diese Studien an, dass die Schadstoffexposition durch Tabakzigaretten im Vergleich weitaus höher läge [35, 36]. Daher muss auch bei der Bewertung immer verglichen werden, wie die gesundheitliche Situation ohne Intervention aussehen würde. Denn bereits die Reduktion der Exposition mithilfe des Umstiegs auf die E‑Zigarette ist so signifikant, dass die E‑Zigarette notwendigerweise bei Rauchstoppprogrammen berücksichtigt werden sollte [7]. Diese Reduktion ist aber nur dann wirklich relevant, wenn der vollständige Umstieg von der Tabak- auf die E‑Zigarette erfolgt.

Hajek et al. [37] haben 2018 eine erste Untersuchung veröffentlicht, die die Effektivität von E‑Zigaretten und verschiedenen NET direkt miteinander vergleicht. In beiden Probandengruppen wurden die Ausstiegsversuche mit den zugeteilten Produkten um verhaltenstherapeutische Maßnahmen wie etwa Gruppentherapien oder Einzelberatungen ergänzt. Die Studienergebnisse zeigen, dass der Konsum von E‑Zigaretten in etwa doppelt so häufig zu einem dauerhaften Rauchausstieg führt wie der Gebrauch von NET. Diese Effektivität lässt sich vermutlich in Teilen darauf zurückführen, dass das Konsumerlebnis der E‑Zigarette als deutlich näher an der ursprünglichen Tabakzigarette empfunden wird, als es bei Nikotinpflastern oder -sprays der Fall ist, und körperliche Entzugserscheinungen dadurch besser ausgeglichen werden. Die Ergebnisse werden jedoch dahin gehend von der Fachwelt kritisch eingeordnet, dass etwa 80 % der Personen, die mithilfe der E‑Zigarette den Tabakkonsum aufgegeben hatten, auch nach einem Jahr noch E‑Zigaretten konsumierten und somit keine vollständige Nikotinabstinenz erreicht wurde [8]. Bei NET war der Prozentsatz mit 9 % der Probanden, die auch nach einem Jahr auf das Produkt (NET) zurückgriffen, deutlich niedriger [37].

Allerdings lässt sich hier wieder darauf verweisen, dass der Nikotinkonsum an sich nach allen wissenschaftlichen Erkenntnissen im Vergleich zum Tabakkonsum oder den im E‑Zigarettendampf enthaltenen Schadstoffen weniger schwere gesundheitliche Folgen hat [7].

Weitere Forschungsergebnisse zeigen auf, dass Nutzer von E‑Zigaretten ähnlich wenige unerwünschte Nebenwirkungen erfahren wie Raucher, die versuchen, mithilfe einer NET aufzuhören [7]. Auch hinsichtlich dieser Variable scheinen NET und E‑Zigaretten also durchaus vergleichbar [7].

Das jüngste Cochrane-Review zur E‑Zigarette bestätigt die Sichtweise, dass E‑Zigaretten möglicherweise genauso effektiv beim Rauchausstieg helfen wie NET [7]. Einzelne vorgestellte Studien weisen sogar eine erhöhte Effektivität der E‑Zigarette bei der Unterstützung eines Rauchausstiegs nach [26]. Trotz aller aktuellen wissenschaftlichen Evidenz, die für E‑Zigaretten und ihr Entwöhnungspotenzial spricht [13], fehlen bisher ausreichend qualitativ hochwertige Studien in ausreichender Anzahl, die die gesundheitlichen Auswirkungen des Konsums von E‑Zigaretten untersuchen [7]. Daher ist die bisherige wissenschaftliche Evidenz noch eingeschränkt zu bewerten, da es auch eine Vielzahl von Studien gibt, die anderslautende Resultate ausweisen [16]. Diese sich noch entwickelnde wissenschaftliche Evidenzlage muss in der öffentlichen wie auch wissenschaftlichen Diskussion berücksichtigt werden.

## Abschließende Diskussion

Es gibt gute wissenschaftliche Argumente, weshalb die bisher von McNeil et al. [15] kommunizierte Einschätzung, dass E‑Zigaretten 95 % weniger schädlich sind, sowohl toxikologisch schwer aufrechtzuhalten ist [38] und als auch aus Sicht der Wissenschaftskommunikation zu kritisieren ist [39]. Dennoch ist es wissenschaftlicher Konsens, dass sich Nutzer von E‑Zigaretten bei normalem Gebrauch weniger Schadstoffen aussetzen, als wenn sie Tabakzigaretten konsumieren [16]. Daher kommt auch der NASEM-Report [16] zu der Einschätzung, dass der komplette Wechsel auf die E‑Zigarette die gesundheitsschädlichen Wirkungen mindestens kurzfristig reduziert. Für Nutzer von Tabakzigaretten verschlimmert der Umstieg auf E‑Zigaretten ihre gesundheitliche Situation nicht, solange kein Dual-use praktiziert wird. Wenn sie das Rauchen komplett durch das Dampfen ersetzen, können E‑Zigaretten eine schnelle und signifikante Reduzierung der individuellen Gesundheitsbelastung darstellen [13, 15].

Das mögliche Potenzial der E‑Zigarette zeigt sich in Deutschland daran, dass es aktuell die beliebteste Variante ist, einen Rauchstoppversuch zu unternehmen, noch vor Nikotinersatzprodukten oder Beratung durch medizinisches Fachpersonal [4]. Dies dürfte auch mit der höheren Verfügbarkeit der Produkte und der gestiegenen öffentlichen Wahrnehmung der Geräte in den vergangenen Jahren zusammenhängen. Gleichzeitig entzündet sich an der steigenden Beliebtheit und Verfügbarkeit auch die wissenschaftliche Diskussion, ob die E‑Zigarette ein sinnvolles Rauchausstiegsinstrument ist [7, 13]. Dennoch haben sich Staaten wie das Vereinigte Königreich [15, 40] oder Neuseeland [41] dafür entschieden, die Potenziale der E‑Zigarette zu nutzen, und sie zu einem weiteren Eckpfeiler ihrer Rauchentwöhnungsprogramme gemacht.

Zwar herrscht aktuell eine lebhafte Debatte in der Wissenschaft, ob und, wenn Ja, inwiefern die E‑Zigarette beim Rauchausstieg hilfreich ist [13], und es gibt gute Gründe für beide Ansichten. Um diese Diskussion weiter evidenzbasiert führen zu können, muss es gelingen, die E‑Zigarette als Produkt und ihre gesundheitlichen Auswirkungen besser zu verstehen [25]. Denn die Anzahl der Studien zur E‑Zigarette und deren gesundheitlichen Auswirkungen wächst zwar kontinuierlich, doch fehlen vor allem qualitativ hochwertige Studien. Dieser Status lässt sich auch für die Wirksamkeit von E‑Zigaretten in der Tabakentwöhnung feststellen. Wie auch Hartmann-Boyce et al. [7] feststellen, gibt es nur eine sehr geringe Anzahl an Studien zur Frage ebendieser Wirksamkeit, die nur ein geringes Verzerrungsrisiko aufweisen. Hier bedarf es also nicht nur eines quantitativen Mehrs an Arbeiten, sondern wissenschaftlich fundierter, randomisiert kontrollierter Studien, um die E‑Zigarette insgesamt, aber auch ihre Rolle bei der Tabakentwöhnung besser zu verstehen [13, 16]. Die Studien, die bereits mit solchen Standards durchgeführt worden sind, deuten jedoch in die Richtung, dass E‑Zigaretten eine sinnvolle Ergänzung eines Rauchstoppversuchs sein können [7] und hier auch etablierten NET möglicherweise überlegen sind. Daher sollte sich die Diskussion stärker an der aktuellen wissenschaftlichen Evidenz orientieren und dem Produkt die Chance gegeben werden, seinen Beitrag für die Tabakentwöhnungspolitik zu leisten.

## Ausblick und Fazit

Dieser Artikel hat aufgezeigt, dass es für den Einsatz der E‑Zigarette bei der Tabakentwöhnung wissenschaftliche Argumente gibt. Der Einsatz sollte auf klar umrissenen und wissenschaftlich fundierten Kriterien basieren. Um diese Kriterien fortlaufend weiterzuentwickeln und an den aktuellen Wissenstand anzupassen, muss auch das Produkt mit seinen Chancen und Risiken besser verstanden werden. Dieser Einschränkung zum Trotz sprechen die aktuell vorliegenden wissenschaftlichen Erkenntnisse nicht dagegen, dass die E‑Zigarette eine größere Rolle bei der Tabakentwöhnung einnehmen sollte als bisher [42]. Daher wäre es begrüßenswert, wenn die nächste S3-Leitlinie zu Rauchen und Tabakabhängigkeit [8] sich verstärkt an dem aktuellen Cochrane-Review [7] zur E‑Zigarette orientieren würde. Dies würde den behandelnden Therapeuten zumindest die Möglichkeit eröffnen, die E‑Zigarette bei der Rauchentwöhnung in Betracht zu ziehen.

Anhand der vorliegenden Forschungsergebnisse muss erneut die Frage gestellt werden, ob man aus gesundheitspolitischen Überlegungen weiterhin direkt eine komplette Nikotinabstinenz erreichen und dabei eine hohe Abbrecherquote riskieren möchte. Alternativ könnte man sich in einem ersten Schritt darauf konzentrieren, die Schadstoffexposition eines jeden Rauchers zu minimieren und erst anschließend einen kompletten Nikotinentzug anzustreben. Da die Zahl der jährlichen Rauchausstiegsversuche in Deutschland seit mehreren Jahren rückläufig ist, besteht dringender Handlungsbedarf hinsichtlich einer Evaluation der aktuellen Tabakentwöhnungsstrategie: Zwischen 2016 und 2019 haben in Deutschland nur etwa 20 % aller Raucher mindestens einen Rauchstoppversuch unternommen. Das bedeutet im Umkehrschluss, dass die überwiegende Mehrheit der Raucher (ca. 80 %) in diesem Zeitraum es erst gar nicht versucht hat, ihre gesundheitliche Situation mit der Aufgabe des Tabakkonsums zu verbessern [4]. Daher sollten alle verfügbaren Werkzeuge eingesetzt werden, wenn nicht grundsätzliche und schwerwiegende Argumente dagegensprechen. Aus Sicht der aktuellen Forschung [7] ist das bei der E‑Zigarette nicht der Fall.

Abschließend soll erneut darauf hingewiesen werden, dass es noch weiterer substanzieller Forschung bedarf, um das genaue Harm-Reduction-Potenzial der E‑Zigarette quantifizieren zu können [16, 21]. Auch vor dem Hintergrund, dass die Geräte und Inhaltsstoffe fortlaufend weiterentwickelt werden, bedarf es einer kontinuierlichen und langfristig angelegten Untersuchung der am Markt vorhandenen Produkte [38].

## Footnotes

1 Für weiterführende Informationen zu COPD: Leitlinie zur Diagnostik und Therapie von Patienten mit chronisch obstruktiver Bronchitis und Lungenemphysem (COPD).

2 *E‑Zigaretten: Das kleinere Übel?* Deutsche Welle. https://www.dw.com/de/e-zigaretten-das-kleinere-%C3%BCbel/a-48843810 (zuletzt abgerufen: 16.09.2021).

3 *Zweifel an Studie zu Gesundheitsgefahr von Dampfen*; ZEIT Online. https://www.zeit.de/wissen/gesundheit/2019-12/e-zigaretten-rauchen-studie-lungenerkrankungen-risiko-wissenschaftler (zuletzt abgerufen: 16.09.2021).

4 *Die Verunsicherung um E‑Zigaretten wächst*; SPIEGEL Online. https://www.spiegel.de/gesundheit/diagnose/e-zigaretten-moegliche-krebsgefahr-die-verunsicherung-waechst-a-1287167.html (Zuletzt abgerufen: 16.09.2021).

5 *Endlich E‑Raucher*; SPIEGEL Online. https://www.spiegel.de/wissenschaft/mensch/e-zigaretten-neue-dampf-zigaretten-sollen-beim-aufhoeren-helfen-a-00000000-0002-0001-0000-000174691266 (zuletzt abgerufen: 16.09.2021).
